# Carbolic Acid as a Local Anæthetic

**Published:** 1871-12

**Authors:** J. H. Bill


Carbolic Acid as a Local Anæsthetic.—Carbolic acid posesses wonderful powers as a local anæsthetic. If a portion of skin is covered with a cloth soaked in a saturated solution of carbolic acid for half an hour, and then a streak traced across the surface with a camel's-hair brush dipped in acid liquefied by one-twentieth its bulk in water, the skin may be divided along the course of the streak with a sharp scalpel, quite down to the subcutaneous cellular tissue, without causing any pain. Abscesses, whitloes, and buboes may be opened with great advantage in this manner. Sometimes it is necessary, when making an incision through the integument, to brush out the wound made with some liquefied acid before making the incision deeper.—*Dr. J. H. Bill*, *Braithwaite's Retrospect.*

